# Exploring the “*anchor word*” effect in infants: Segmentation and categorisation of speech with and without high frequency words

**DOI:** 10.1371/journal.pone.0243436

**Published:** 2020-12-17

**Authors:** Rebecca L. A. Frost, Kirsty Dunn, Morten H. Christiansen, Rebecca L. Gómez, Padraic Monaghan

**Affiliations:** 1 Max Planck Institute for Psycholinguistics, Nijmegen, Netherlands; 2 Lancaster University, Lancaster, United Kingdom; 3 Cornell University, Ithaca, New York, United States of America; 4 University of Arizona, Tucson, Arizona, United States of America; CNRS - Université d’Aix-Marseille, FRANCE

## Abstract

High frequency words play a key role in language acquisition, with recent work suggesting they may serve both speech segmentation and lexical categorisation. However, it is not yet known whether infants can detect novel high frequency words in continuous speech, nor whether they can use them to help learning for segmentation and categorisation at the same time. For instance, when hearing “*you eat the biscuit*”, can children use the high-frequency words “*you*” and “*the*” to segment out “*eat*” and “*biscuit*”, and determine their respective lexical categories? We tested this in two experiments. In Experiment 1, we familiarised 12-month-old infants with continuous artificial speech comprising repetitions of *target words*, which were preceded by high-frequency *marker words* that distinguished the targets into two distributional categories. In Experiment 2, we repeated the task using the same language but with additional phonological cues to word and category structure. In both studies, we measured learning with head-turn preference tests of segmentation and categorisation, and compared performance against a control group that heard the artificial speech without the marker words (i.e., just the targets). There was no evidence that high frequency words helped either speech segmentation or grammatical categorisation. However, segmentation was seen to improve when the distributional information was supplemented with phonological cues (Experiment 2). In both experiments, exploratory analysis indicated that infants’ looking behaviour was related to their linguistic maturity (indexed by infants’ vocabulary scores) with infants with high versus low vocabulary scores displaying novelty and familiarity preferences, respectively. We propose that high-frequency words must reach a critical threshold of familiarity before they can be of significant benefit to learning.

## Introduction

For the majority of humans, linguistic proficiency is attained with remarkable ease. Yet, in order to get there, learners must develop a broad range of complex skills—including finding individual words in running speech, and figuring out how to recognise which grammatical categories those words belong to in order to interpret (and later, convey) meaning. Although speech contains no absolute cues for either word segmentation (e.g., [1]) or grammatical categorisation (e.g., [2]), one cue that has been suggested to aid learning for both of these tasks is the distribution of information in speech; the way in which particular phones and syllables co-occur can provide a strong indication of what constitutes words in a given language, while commonalities among the phonological properties of those words [3, 4] and information about the way words are used in combination [2, 5] can provide a helpful description of the categories those words belong to.

Infants have a striking sensitivity to the distributional information contained in speech (e.g., [6]), and are capable of detecting co-occurrence information for use on a range of language-learning tasks—from speech segmentation (e.g., [7]), to lexical categorisation [8–10], and acquisition of syntax-like constraints (e.g., [11, 12]). This statistical sensitivity is suggested to emerge at an early age [6]—perhaps even from birth [13]—and thus may play a key role in infants’ early language acquisition. From around 8 months, infants can use syllable-transition probabilities to segment speech into individual words (e.g., [7, 14]). At around the same age, infants can detect simple distributional structure in speech (such as AAB/ABA structures, e.g., [11, 15, 16], see also [17]), with this skill possibly increasing in sophistication over development (see e.g., [18–22]). Soon after (from around 12 months), infants can also use distributional information (such as word co-occurrence, and phonological regularities) to guide their formation of abstract lexical categories [8–10, 19, 23].

Infants are therefore well equipped to draw on the rich distributional landscape of speech to help them during language acquisition. With this in mind, it is perhaps unsurprising that infants have also been shown to draw on item frequency—as well as item co-occurrence—during learning. Frequency has been found to play an important role in language acquisition, with infants learning frequent morphemes, words, and syntactic constructions significantly earlier than their less frequent counterparts (see e.g., [24], and see [25] for a review). Words occurring with high frequencies have been found to be particularly beneficial for infants’ language learning, as they provide more reliable co-occurrence information than words which occur less often [4], and are more easily perceived than lower-frequency words of a similar length [26, 27]. Recent research has suggested that high-frequency words may also benefit language acquisition in another key way; by assisting with speech segmentation [28–32].

Highly frequent words have been suggested to help speech segmentation by signposting the boundaries of the words that surround them in speech–operating as *anchor* points, which further speech segmentation can occur around [29]. One intriguing possibility is that this “anchor effect” may help learning by facilitating interplay between top-down lexical segmentation (drawing on learners’ existing knowledge of words), and bottom-up identification of the edges of unfamiliar items (drawing on the statistics of the input), thereby helping learners to identify both familiar and unfamiliar words in running speech (e.g. [33, 34]). Take, for example, the sentence *you eat the biscuit yet you drink the milk*. When an infant hears this sentence, they may recognise high frequency words *you* and *the*, and use these to help uncover the words that surround them in speech. Yet, some parts of the utterance will remain unsegmented (*biscuit yet*), so infants must look to additional sources of information in order to tease these words apart, such as the transitional probabilities of syllables within words (e.g., [6], or the many phonological [35] or prosodic cues that have been suggested to support segmentation [36–40].

The notion that elevated word-frequency benefits speech segmentation has gained notable traction in the language development literature. The first empirical evidence for this was observed by Bortfeld et al. [29] who found that 6-month-old infants were better at segmenting new words from speech when they were presented alongside words that were already highly familiar (such as the infant’s own name, or the word ‘mommy’), compared to when they appeared alongside another new word. Subsequent research has since provided compelling support for this anchor effect for both infant [31, 32, 41] and adult [34] learners, with recent key evidence coming from Cunillera, Laine, and Rodriguez-Fornells, who documented the neural signature of this effect—with anchor words eliciting greater stimulus-preceding negativity (a marker of expectation for subsequent input) in adults’ EEG than their less frequent counterparts [42].

Further support for the anchor word effect can be found in computational modeling literature. Monaghan and Christiansen [43] devised a model of speech segmentation (PUDDLE) which operated by treating each utterance as a potential word, and segmenting utterances when they contained previously identified word candidates. When applied to natural language corpora of child-directed speech, the model quickly extracted high-frequency words, and used them to help segment the rest of the speech input. When viewed in combination with the behavioural data, these findings provide converging evidence that high-frequency words may assist early language acquisition by facilitating speech segmentation. Prior research on a similar corpus with child-directed speech sheds interesting light on the nature of these high frequency words, demonstrating that function words occurred with far greater frequencies than other items, constituting the entirety of the 10 most frequent words out of a corpus of around 2.6 million words (*“you”* = 124219; *“the”* = 81029; *“it”* = 59629; *“a”* = 56952; *“to”* = 51760; *“I”* = 50418; *“what”* = 48081; *“that”* = 43202; *“and”* = 41780; *“is”* = 34513), with even relatively frequent content words occurring far less often (e.g., *“mummy”* = 1510; *“play”* = 4096; *“eat”* = 3960; “*drink”* = 1017; *“sleep”* = 822; *“nappy”* = 70, *“diaper”* = 162, *“dummy”* = 33, *“pacifier”* = 6; [44]). High frequency words (predominantly function words) may, therefore, play a particularly important role in segmenting speech.

In a recent study with adults, Frost, Monaghan, and Christiansen [45] examined the possibility that the benefit of high frequency words may actually be twofold—with these items potentially assisting with the *categorisation* of new words, in addition to helping with their initial discovery [46–49]. This was in light of the substantial overlap observed between the high frequency words that were seen to assist segmentation in the PUDDLE model [43] and words that were found to cue grammatical categorisation in prior corpus analyses [2] which indicated that the same items could conceivably inform learning for both tasks at the same time. We can see how this may work with the example sentence “*you eat the biscuit yet you drink the milk*”—*you* reliably precedes verbs (*eat*, *drink*), while *the* reliably precedes nouns (*biscuit*, *milk*), in keeping with Mintz’ [47] observation that in English, pronouns and determiners reliably precede verbs and nouns, respectively—potentially cueing grammatical categorisation.

Frost et al. [45] examined this possibility by training adults on an artificial language comprising lower frequency bisyllabic target words and high frequency mono-syllabic marker words, which distinguished target words into two otherwise unidentifiable categories (with one marker word reliably preceding targets in each category). After exposure to a continuous speech stream, participants were tested on their ability to segment the speech into words, and form distributional categories based on marker-target word co-occurrence. Performance was compared to that of a control condition who were trained on a language that comprised target words only. Speech segmentation scores were similar for both groups, but the marker words shaped adults’ formation of grammatical categories, suggesting that these high-frequency words may inform categorisation during the early stages of language acquisition–perhaps while learners are still discovering how to segment speech [45].

A critical test of the way that high frequency words impact segmentation and categorisation during early language acquisition would be to examine the way that they influence learning in infants. Yet, to date, no study has examined these tasks together. However, we know that infants can draw on statistical information to segment speech (e.g., [6]), and make use of highly frequent items to help locate boundaries for new words [29, 31, 41]. Similarly, we know that infants can also draw on distributional information in speech to divide new words into abstract categories [8–10]. Thus, it is conceivable that infants may be able to draw on high frequency words to help with both of these tasks during learning.

However, combining short high frequency words and longer, lower frequency words in speech means that children will be faced with the challenge of segmenting words of different lengths. This is a key feature of natural language, and while children can undoubtedly cope with this ‘in the wild’, examining how they do so has proven challenging in prior research, and whether it is possible under laboratory conditions for artificial language remains an open question (though see [45] and [50] for findings that suggest this is possible for adults). In fact, Johnson and Tyler [51] proposed that infants’ ability to segment speech was limited, such that words had to be of similar length in order for statistical segmentation to proceed. Five- and 8-month old children were trained on a continuous artificial language comprising either only bisyllabic words or bisyllabic and trisyllabic words. Children learned to identify words in the bisyllabic language, but not the language with varying lengths. Similarly, Wang, Zevin, and Mintz [52] proposed that early stages of language learning are only possible if the structure to be acquired is regular and rhythmical–if word length varies, then Wang et al. [52] predict that learning will not be successful. Thus, prior findings offer a mixed account of whether infants can indeed segment words of varying lengths from speech using statistical regularities alone. Understanding the limits of infants’ capacity for statistical segmentation is vital for constraining theorising about the way in which it proceeds during language acquisition.

We test whether infants can segment speech comprising words of different lengths when these correspond to alternations between high-frequency (monosyllabic markers) and low-frequency words (bisyllabic targets), as is the case in natural language (e.g., [44]). In Experiment 1 we examine whether 12-month-olds’ speech segmentation and distributional categorisation is shaped by the presence of high frequency words. We hypothesised that high-frequency words operating as markers to word boundaries might assist with speech segmentation [28–32]. Additionally, we hypothesised that these marker words might also contribute to infants’ formation of grammatical categories [8, 9, 19, 23].

In Experiment 2 we examine the effect of high frequency words in combination with additional phonological cues, which have been suggested to play a vital role in speech segmentation (e.g., [35]) and categorisation [3, 53, 54]–particularly when they occur in combination with other distributional cues [4, 8, 10, 19, 23, 55, 56]. We expected that learning would be best when infants were trained on a language containing phonological cues in addition to the high frequency words.

In both studies, in exploratory analyses, we also investigate whether children’s looking preferences at test are related to their linguistic maturity. In previous work, it has been shown that children’s language proficiency relates to their ability to process the statistical structure of artificial languages [57] and also relates to whether infants respond with greater interest to novel or habituated items after being exposed to a continuous artificial language [58]. These studies suggest that looking behaviour at test is dynamic, and may reflect something meaningful about the linguistic competence of the learner, or the degree to which information has been learned (e.g., [58–60], and see also [61]).

## Experiment 1: Can 12-month-olds segment and categorise speech containing high frequency words?

### Method

#### Participants

Participants were 32 infants (18 boys, 14 girls), aged between 11.5 and 12.5 months (mean age = 357 days), recruited from Lancaster, Lancashire UK. All infants were monolingual native English learners, born at term, with normal vision and hearing, and were typically-developing at the time of testing. Infants were tested in the laboratory at Lancaster University. The study was approved by the Faculty of Science and Technology Research Ethics Board at Lancaster University (FST16168), and was carried out in accordance with the World Medical Association Declaration of Helsinki. All parents/caregivers gave written informed consent prior to their infant’s participation in the study.

#### Design

The experiment used a between-subjects design, with two conditions of training type; *No Markers* (N = 16; boys = 8, girls = 8) and *Markers* (N = 16; boys = 10, girls = 6). These conditions varied the way that marker words were used in the training speech, and either contained no marker words, or two (one marker word per category). Infants were pseudo-randomly allocated to one of these conditions. Knowledge of the experimental language was tested immediately after training using an adaptation of the head-turn preference paradigm (detailed below), with all infants first completing speech segmentation trials, followed by distributional categorisation trials. The experimental language and the stimuli and procedure for each of these tasks are outlined below.

#### Materials

*Stimuli*. Speech stimuli were created using the Festival speech synthesiser [62]. Six *target words* were created from a pool of 12 consonants (*b*, *p*, *d*, *k*, *t*, *g*, *w*, *r*, *l*, *j*, *m*, *n*), and 5 vowels (*a*, *e*, *i*, *o*, *u*) which were combined pseudo-randomly to create bisyllabic CVCV words (e.g., *lupi*, *jedo*, *kuwa*, *bimo*, *garu*, *nute)*. Plosive and continuant consonants and front and back vowels were distributed equally across target words (and across positions within words) to ensure there were no phonological cues to word structure or category membership. Two monosyllabic *marker words* were created from two additional consonants and vowels (*v*, *z*, *i*, *ae*, giving e.g., *vi*, *zhae*), and these preceded target words in the speech stream. There was no repetition of vowels or consonants within target words. Each target word lasted approximately 500ms, and each marker word lasted approximately 250ms. Four transitions between words were omitted from the familiarisation streams (so, did not occur in the training speech), in order to create a set of non-words involving syllable transitions that were not heard during training for either condition (TPs all = 0, see Segmentation Test for more details).

The six target words were arbitrarily split into two equal categories (*A* and *B*), with three words in each category. Category membership was denoted only by the co-occurrence of target words and marker words in the speech stream: in the Markers condition, one marker word reliably preceded words from each category (e.g. *vi* preceded A words, whereas *zhae* preceded B words). The speech stream for the No Markers condition contained target words only, meaning infants in this condition received no information regarding the category membership of the target words (so, we would not expect them to demonstrate such knowledge at test).

Four versions of the language were created and counterbalanced across participants, to ensure that any learning effects observed were not due to infants’ preference for certain combinations of syllables [63]. For each version, target words were created by generating syllables from the pool of consonants/vowels, then pseudorandomly concatenating these into words in line with the criteria outlined above (i.e., with no reliable phonological cues within words, or within categories). Marker words always comprised the same consonants and vowels, and were either vi and zhae, or zhi and vae.

*Training*. A continuous stream of synthetic speech was created using the Festival speech synthesiser [62] by concatenating target words and marker words (see Table 1). For the No Markers condition, the speech stream comprised target words only, and lasted approximately 9 minutes. For the Markers condition, the speech stream comprised target words plus two marker words, and lasted approximately 14 minutes. This duration is in line with the standard procedure for incidental exposure (see e.g., [64], and note that target words were presented with equal frequencies in both conditions). The speech stream was produced using a female voice at 200 Hz with no immediate repetition of individual target words, and speech was continuous—with no pauses between words. The speech stream had a 5 second fade in and out so that the onset and offset of speech would not cue word boundary identification. Note that interspersing high-frequency marker words among bisyllabic target words introduces further variation in syllable-transition probabilities (while within-word TPs for targets were always 1, between-word TPs varied across the conditions; for the no marker condition, forward TPs between targets ranged between 0.2 and 0.25, whereas for the marker condition, TPs were 0.33 between markers and targets, and 0.5 between targets and the following marker word). In natural language, high-frequency marker words also exert this effect on the transitional probabilities in speech, and so effects on segmentation performance may be due to recognition and use of the high-frequency word as a boundary marker, and/or due to effects arising from variation in TPs. As these effects are coexistent in natural language, we do not distinguish these effects in this experiment.

**Table 1:** Example speech streams for each condition in experiment 1.

	Target words	Marker words	Speech Stream Excerpt
No Markers	*lupi*, *jedo*, *kuwa*, *bimo*, *garu*, *nute*		*…bimo-**lupi*-*jedo**-garu-**kuwa**-nute-**jedo**-bimo-**lupi**…*
Markers	*"*	***vi***, ***zhae***	*…****zhae****-bimo-****vi***-*lupi*-***vi***-*jedo*-***zhae****-garu-****vi***-*kuwa*-***zhae****-nute-****vi***-*jedo*-***zhae****-bimo-****vi***-*lupi**…*

*Note*. Items that are underlined belong to Category A, whereas items that are not underlined belong to Category B. Bold items with and without an underline are the marker words for the Category A and Category B targets, respectively. Dashes indicate word boundaries, but these were not physically denoted in the continuous speech; items followed each other directly with no pauses between words.

*Segmentation test*. We assessed segmentation by measuring looking times to two types of trial; *words* and *non-words*. Each *word* trial comprised repetitions of one of the words used in the familiarisation stream (e.g., *lupi*, *lupi*, *lupi*…). *Non-word* trials contained repetitions of items created from the four withheld transitions detailed above, comprising the last syllable of one word and the first syllable of another (e.g., *piku*, *piku*, *piku*…). There were four word trials and four non-word trials, giving eight segmentation trials in total.

We used non-words (items which did not occur during familiarisation) to permit comparison across the different conditions. For the No Markers condition, particular transitions between target words were withheld from the speech stream, and the non-words were formed from the resulting syllable transitions (so for this group non-words are comparable to part-words in classic segmentation tests). The same non-words were used for the Markers condition, and these did not occur in the familiarisation speech for an additional reason—because a marker word intervened the target words. Note that it would not have been possible to use part-words which spanned word boundaries as in Saffran et al.’s [6] studies of speech segmentation, since part-words did not occur in a comparable way across conditions: Part-words in the Markers condition would comprise a fragment of a target word and a marker word, whereas in the No Markers condition they would comprise fragments of two target words (see Frost et al., [45], for an analogous 2AFC task with adults).

*Categorisation test*. We assessed categorisation by measuring looking times to two types of trial; *consistent* and *inconsistent*. *Consistent* trials comprised repetitions of two words from the same distributional category (as determined by their co-occurrence with particular marker words in the familiarisation stream, e.g., *lupi*, *jedu*, *lupi*, *jedu*…). *Inconsistent* trials contained repetitions of two words from different distributional categories (so, they occurred in the training speech with different marker words; e.g., *lupi*, *bimo*, *lupi*, *bimo*). There were four consistent trials and four inconsistent trials, giving eight categorisation trials in total. (see Frost et al., [45], for an analogous task with adults).

#### Procedure

Infants were familiarised with the experimental language via incidental learning [64], with infants playing quietly with the experimenter (with no verbal communication) while the speech stream played at a comfortable volume in the background. During the incidental learning phase, caregivers filled out the UK-CDI [65].

Following familiarisation, we assessed infants’ learning using an adaptation of the classic head turn preference paradigm [66]; each test-item was presented with a visual stimulus which appeared on the left or right of the screen, and we measured infants’ looking times to each test trial–with a difference in looking times to each type of trial indicating learning. Eye movements were coded online by the experimenter using E-prime, which automatically calculated infants’ looking times (analogous to Habit X, [67]; for similar methodology see e.g., [6, 12, 68]. To ensure accuracy with coding, the experimenter received thorough training for this study in online and offline coding sessions, and was naïve to the nature of the test trials. Coding was performed in a private section of the lab, separated from the infant and their caregiver by a walled curtain. Infants were seated on their caregiver’s lap 50–70 cm away from a 21.5 inch 1,920 x 1,080 computer screen.

Sound stimuli were played through speakers positioned behind the monitor to the left and right sides of the screen. Test items were paired with the same visual stimulus (a growing and shrinking rainbow pinwheel) set against a black background, which appeared onscreen on either the left or the right, in accordance with the location of the sound. Presentation of *word* and *non-word* segmentation trials, and *consistent* and *inconsistent* categorisation trials was controlled such that half of each type of trial appeared to the left, and the other half to the right. On each trial infants heard repetitions of a test-item separated by a 500ms pause, and items played in the same voice and at the same rate as in familiarisation. Trials could last for a maximum of 65 seconds (see [21, 22]) and were contingent on infants’ looking behaviour, such that trials automatically terminated if an infant looked away from the visual stimulus for more than 2 seconds. After each trial ended, a fixation stimulus appeared at the centre of the screen, and the next trial began after infants had redirected their attention to the screen. Infants completed the segmentation trials first followed by the categorisation trials (so as to prevent exposure to isolated words on the categorisation task from impacting performance on segmentation trials). Trial order within the tasks was randomised across participants.

## Results & discussion

### Data preparation

Filtering criteria were applied to the data prior to analysis: For the segmentation task, trials with looking times shorter than 500 ms (the approximate length of a test item) were excluded from analysis, as were trials with looking times greater than 2SD beyond the mean looking time for that trial. For the categorisation task, trials shorter than 1500 ms (the approximate time taken to hear both test items) were excluded from analysis, as were trials greater than 2SD beyond the mean for that trial. For each infant, we enforced a minimum inclusion criterion of one trial per type, to permit comparison across the types of trials. That is, if infants only provided data for one type of trial (after the data were filtered), then they were excluded from the analysis.

### Segmentation

On average, infants looked similarly to each type of trial, with infants looking to *word* trials for *M*_raw_ = 7486.653 ms (*SE*_raw_ = 623.292), and to *non-word* trials for *M*_raw_ = 7649.525 ms (*SE*_raw_ = 552.717).

Infants’ looking times were log transformed to account for skewness (determined through visual inspection of histograms and QQ plots and through the Shapiro-Wilk normality test; W = .817, *p* < .001), and all analyses were performed on the log transformed data. The data were analysed in R (4.0.2 [69]) using linear mixed-effects models [70], which were computed with the lme4 package (1.1.21 [71]), modelling the probability (log odds) of looking times considering variation across participants and materials, and across the two types of test items (words and non-words). P values were computed using lmerTest (3.1.2, [72]), and 95% CIs were calculated using the coef function in R. Semi partial R^2^ were calculated for individual main effects and interactions using the r2glmm package (0.2.1, [73]; calculated using the Kenward-Roger approach, as recommended for small samples, and given for effects that are significant, or that are approaching conventional thresholds of significance). A summary of the final model is reported in Table 2.

**Table 2:** Summary of the linear mixed-effects model of (log transformed) looking times on the segmentation trials for participants in experiment 1.

Fixed effects	Estimated coefficient	*SE*	Wald confidence intervals		*t* value	*p* value (*t*)
			2.50%	97.50%		
(Intercept)	8.629	.17	8.296	8.962	50.798	< .001
Word type	-.03	.038	-.104	.044	-.797	.432
Markers condition	-.128	.104	-.332	.075	-1.234	.227
Word type * markers condition	-.012	.038	-.086	.062	-.313	.756
Random effects	Variance	Std. Dev.				
Subject (Intercept)	.305	.553				
Subject:word type (slope)	.008	.092				
Trial (Intercept)	.145	.38				

236 observations, 32 participants, 8 trials. R syntax for the final model is: lmer (logtotallooking ~ word_type*markers_condition + (1+word_type|subject) + (1|trial), data = seg_no_phono, REML = TRUE, control = lmerControl(optimizer = "bobyqa",optCtrl = list(maxfun = 1e+9))).

The model contained fixed effects and interactions for word type and markers condition, and was initially fitted with random intercepts of subject, trial (1–8), presentation version (A or B, with version A beginning with a *word* trial, and B beginning with a *non-word* trial), stimuli location (left or right), and item, with a nested random slope of language version (1–4). We sought to fit the maximal random effects structure as justified by the experimental design [74]. If the model failed to converge, the random effects structure was simplified until convergence was no longer an issue.

**Fig 1:**
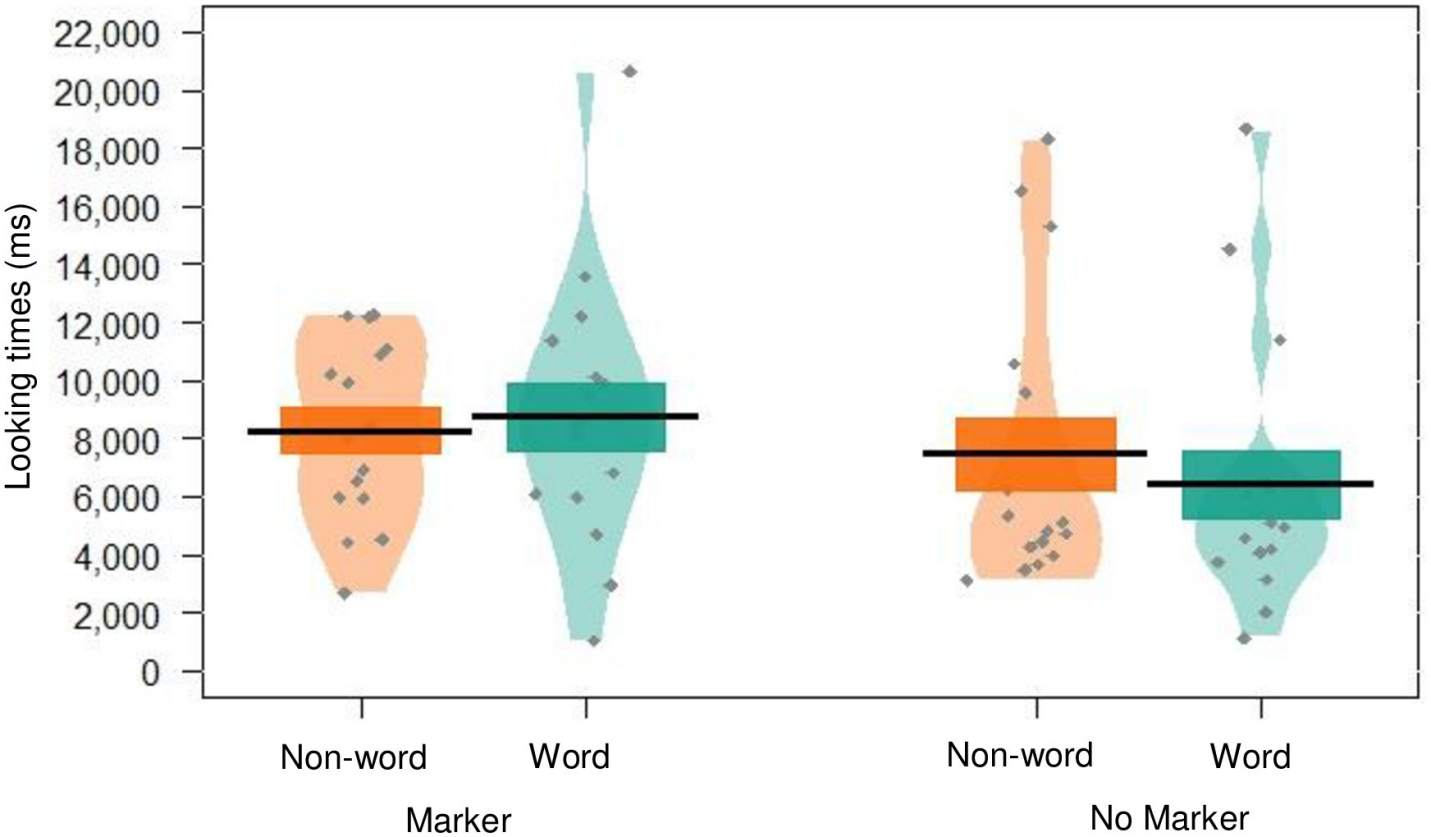
Pirate plot depicting the (raw) mean looking times to words and non-words, given for each condition. Black lines indicate the mean, and coloured blocks indicate *SE*. The distribution of looking times is illustrated for each group, with individual data points in grey.

There was no effect of word type, with infants looking similarly to non-word and word trials (*p* = .432). There was also no effect of markers condition (*p* = .227), and no significant interaction between markers condition and word type (*p* = .756, see Fig 1), suggesting that looking to words versus non-words was not mediated by the presence or absence of markers in the speech stream. Thus, there was no evidence that infants were able to segment the speech stream, and there was no evidence to suggest that they relied on the marker words during learning.

#### Exploring looking preferences and vocabulary size: Segmentation

In subsequent exploratory analysis, we examined learning as a function of language proficiency, to establish whether infants’ looking behaviour was shaped by their linguistic maturity [58, 60]–indexed here by their scores on the UK CDI [65]. To this end, we performed a median split on the data according to infants’ receptive CDI scores; those with a score of 63 or higher were classified as *High-Vocabulary* infants, whereas those with a score of 62 or below were classified as *Low-Vocabulary* infants. We performed linear mixed-effects analysis on the data, with the critical tests being the interactions involving vocabulary size, word type, and markers condition. The models were built in the same way and with the same random effects structure as those described above. A summary of the final model is reported in Table 3.

**Table 3:** Summary of the linear mixed-effects model of (log transformed) looking times on the segmentation trials for participants in experiment 1 (with median split for vocabulary size).

Fixed effects	Estimated coefficient	*SE*	Wald confidence intervals		*t* value	*p* value (*t*)
			2.50%	97.50%		
(Intercept)	8.637	.111	8.42	8.854	77.939	< .001
Word type	-.041	.063	-.164	.082	-.649	.539
CDI score	.152	.102	-.047	.351	1.495	.147
Markers condition	-.145	.102	-.344	.055	-1.422	.167
CDI score*Word type	.088	.045	.001	.176	1.976	.051
CDI score*Markers condition	-.124	.102	-.323	.075	-1.218	.234
Word type*Markers condition	-.005	.045	-.093	.083	-.108	.914
CDI score*word	-.04	.045	-.127	-.048	-.886	.378
type*Markers Condition						
Random effects	Variance	Std. Dev.				
Subject (Intercept)	.259	.509				
Subject: word type (slope)	.004	.067				
Item (Intercept)	.016	.125				

228 observations, 31 participants, 8 trials. R syntax for the final model is: lmer (logtotallooking ~ cdi_score*word_type*markers_condition + (1+word_type|subject) + (1|item), data = seg_no_phono_complete, REML = TRUE, control = lmerControl (optimizer = "bobyqa", optCtrl = list (maxfun = 1e+9))).

**Fig 2:**
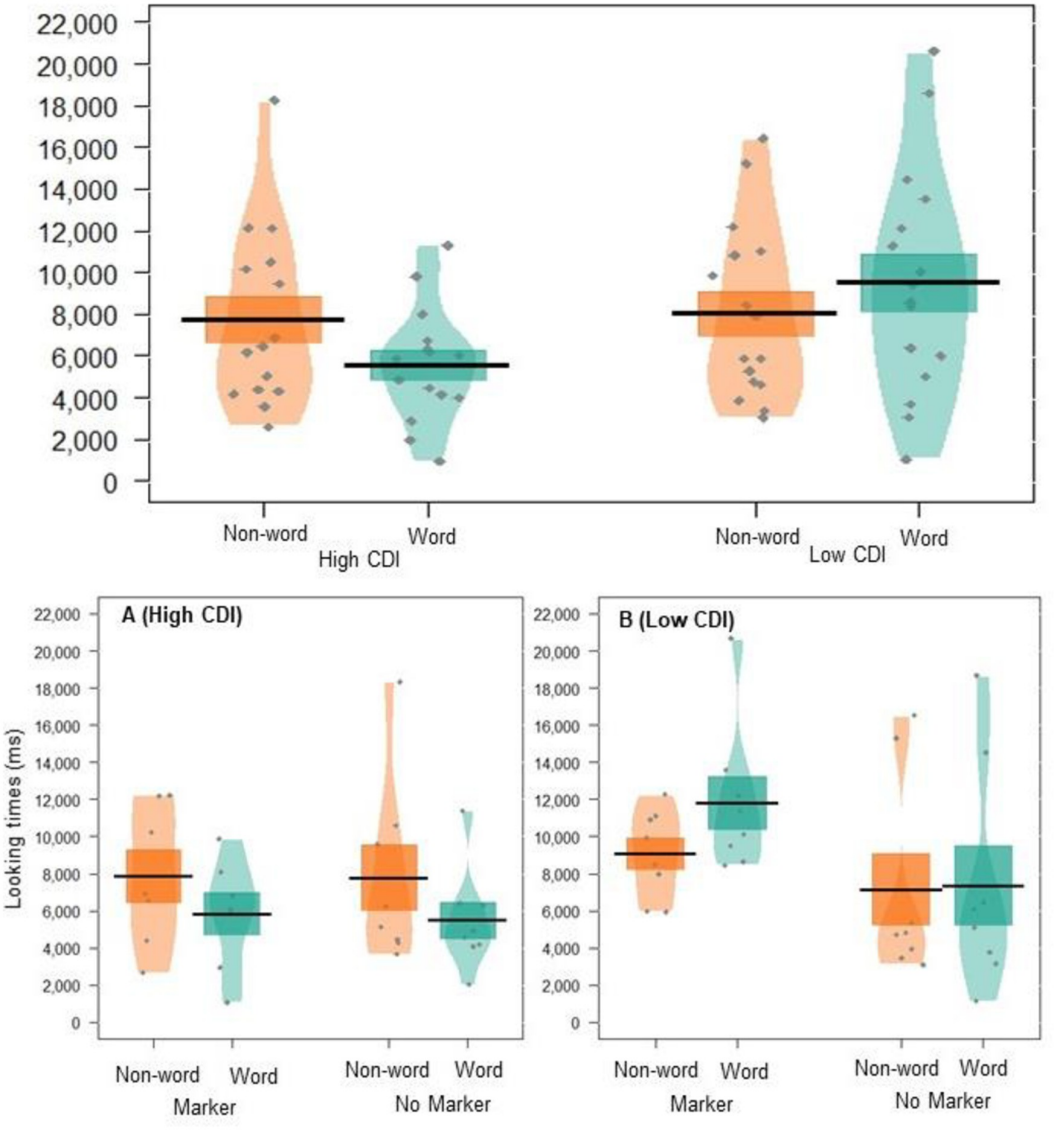
Pirate plot depicting the (raw) mean looking times to words and non-words for the participants in experiment 1. The top panel displays this data for High and Low CDI groups. The bottom panel breaks this down into each markers condition, with the High CDI group in panel A and the Low CDI group in panel B. Black lines indicate the mean, and coloured blocks indicates *SE*. Coloured shapes show the distribution of looking times for each group, with individual data points in grey.

There was no effect of vocabulary size on overall looking times (*p* = .147). However, the interaction between vocabulary size and word type was approaching significance (*p* = .051, *semi partial $R_{m\;}^{2}=.13$*); high-vocabulary infants had a novelty preference, looking longer at non-words (*M*_raw_ = 7483.887, *SE*_raw_ = 888.012) than words (M_raw_ = 5477.855, *SE*_raw_ = 529.748), whereas low-vocabulary infants had a familiarity preference, looking longer at words (*M*_raw_ = 9549.288, *SE*_raw_ = 1082.154) than non-words (*M*_raw_ = 7998.033, *SE*_raw_ = 734.504; see Fig 2). This suggests that infants may have been trending towards segmentation (though this effect did not reach statistical significance–see the results described above), and that their looking preferences were mediated by their language development. These differences in looking preferences for low versus high CDI infants are in line with the prior suggestion that infants’ looking preferences are shaped by their linguistic maturity, with novelty preferences depicting a more mature response [60]. Higher powered replications are required to confirm these results. The three-way interaction between vocabulary score, word-type, and markers group was not significant (*p* = .378).

### Categorisation

On average, infants looked to *consistent* trials for *M*_raw_ = 4089.024 ms (*SE*_*r*aw_ = 321.01), and to *inconsistent* trials for *M*_raw_ = 3804.562 ms (SE_raw_ = 221.691). Trends in the means indicate no overall difference in looking to each type of trial at the group level.

Infants’ looking times were log transformed to account for skewness (determined through visual inspection of histograms and QQ plots, and through the Shapiro-Wilk normality test; W = .786, *p* < .001), and linear mixed-effects analysis was performed on the transformed data. The model contained fixed effects and interactions for trial type (*consistent* and *inconsistent*) and markers condition, and was initially fitted with a maximal random effects structure [74], with random intercepts of subject, trial (1–8), presentation version (A or B, with version A beginning with a *word* trial, and B beginning with a *non-word* trial), stimuli location (left or right), and item, with a nested random slope of language version (1–4). If the model failed to converge, the random effects structure was simplified until convergence was no longer an issue. The critical result here was the interaction between markers condition and trial type; if infants can draw on the high frequency words to help categorise the targets, then we should see evidence of this for the markers group, however we should see no categorisation for the no marker group since they did not receive any cues to category membership.

**Fig 3:**
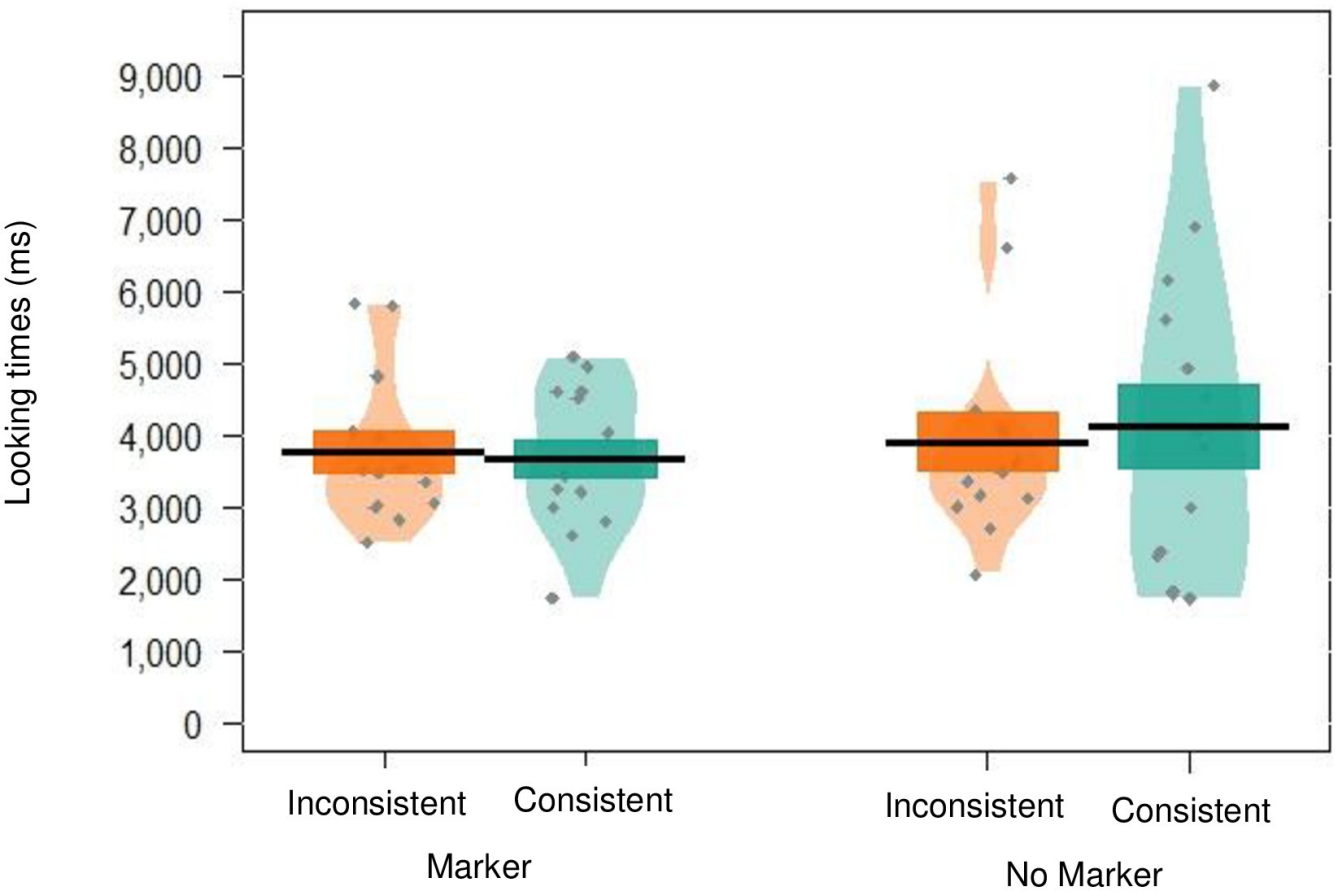
Pirate plot depicting the (raw) mean looking times to trials containing words from the same (*consistent*) versus different (*inconsistent*) categories for participants in experiment 1, given for each condition. Black lines indicate the mean, and the coloured blocks indicate *SE*. The distribution of looking times is given for each group, with individual data points in grey.

There was no significant effect of trial type (*p* = .887), indicating that infants looked similarly to trials containing words from the same category and trials containing words from different categories. There was no significant effect of markers condition (*p* = .876), and the interaction between word type and markers condition was also not significant (*p* = .523), suggesting there was no difference in looking across the conditions (see Fig 3, and see Table 4 for a summary of the full model). These data therefore suggest that infants did not form distributional categories on the basis of co-occurrence between targets and the high frequency markers.

**Table 4:** Summary of the linear mixed-effects model of (log transformed) looking times on the categorisation trials in experiment 1.

Fixed effects	Estimated coefficient	*SE*	Wald confidence intervals		*t* value	*p* value (*t*)
			2.50%	97.50%		
(Intercept)	8.128	.086	7.96	8.296	94.405	< .001
Markers condition	-.007	.044	-.093	.079	-.158	.876
Trial type	.006	.041	-.075	.087	.143	.887
Markers condition*Trial Type	.026	.04	-.053	.104	.642	.523
Random effects	Variance	Std. Dev.				
Subject (Intercept)	.012	.108				
Subject: trial type (slope)	.002	.045				
Trial (Intercept)	.006	.076				
Lang_version (Intercept)	.009	.094				
L_or_R (Intercept)	.005	.505				

172 observations, 28 participants, 8 trials. R syntax for the full model is: lmer (logtotallooking ~ trial_type*markers_condition + (1+trial_type|subject) + (1|lang_version) + (1|trial) + (1|L_R), data = anchors12_cat_filt1500np, REML = TRUE, control = lmerControl (optimizer = "bobyqa", optCtrl = list(maxfun = 1e+9))).

#### Exploring looking preferences and vocabulary size: Categorisation

Supplementary exploratory analysis was performed on the categorisation data, to examine whether infants’ looking behaviour was shaped by their linguistic maturity. Linear mixed effects analysis were performed on the (log transformed) data to test for differences in looking behaviour for high vs. low vocabulary infants (using the same median split criteria, and the same random effects structure described above). All effects and interactions involving CDI score were not significant (all *p* > .18, see the supplementary materials (provided on OSF) for a full summary of this model).

## Experiment 2: Do high frequency words impact language learning when combined with additional phonological cues?

In Experiment 1, contrary to our expectations, there was no clear evidence to suggest infants segmented the words from speech in either the experimental or control condition, and high frequency words were not seen to benefit learning. Infants also failed to draw on the co-occurrence between high frequency marker words and target words to form distributional categories. In Experiment 2 we examine how infants’ learning proceeds from input containing high frequency words in combination with additional phonological cues–which may critically assist learning.

It is well established that infants draw on myriad sources of information during language learning (e.g., [75, 76]), and it is possible that infants require additional cues in order to succeed on the tasks in the study at hand (e.g., [8, 23]). We addressed this possibility by incorporating within-word and within-category phonological regularities into the artificial language, which cued word identification and category membership respectively. Specifically, target words were formed from two CV syllables that shared the same acoustical phonological properties (i.e., plosive consonants and front vowels, or continuant consonants and back vowels, with four words of each type), with within-word harmony among both consonants and vowels cueing word identification. In addition, all words in the *A* category contained front vowels and plosive consonants, whereas all words in the *B* category contained back vowels and continuant consonants, cuing categorisation.

Phonological cues have been suggested to play a pivotal role in early language learning; well within their first year of life, infants become highly attuned to the phonological regularities in their native language (see e.g., [77]), and can use this information to help with acquisition of both words and syntax [78–80]. With regard to word identification and speech segmentation, learners have been found to draw on word-internal phonological regularities such as vowel harmony (where vowels within words are all the same type, e.g., [81–83]) and commonalities or restrictions among use of particular consonants or consonant types [63, 84–88]; see [89], for a review). Such regularities among vowels [35, 90–92] and consonants [93, 94] have been found to help learners discover words in speech from early infancy onward, and thus may provide valuable support for speech segmentation in the current study.

Similarly, the phonological properties of words have been found to have a profound effect on lexical categorisation, with similarity between words belonging to the same lexical category significantly influencing learning (e.g., [3, 53, 54, 95]. Note that we focus on the distribution of vowels and consonants as cues to lexical categories, but see Monaghan, et al., [2] for an overview of the many other types of phonological cues that may assist learning). Correspondence between phonological features and grammatical categories has been noted for a number of languages—for instance, Sereno and Jongman [96] demonstrated that in English, for highly frequent nouns and verbs, the phonological structure of words is distinct, such that nouns are more likely to have front than back vowels, while the opposite is true for verbs (see also [3, 4] and see Shi et al., [56] for noun/verb phonological distinctions in Turkish and Mandarin, and Monaghan et al., [2], for work on English, Dutch, French, and Japanese). By 12 months, infants can use these phonological distinctions to form simple lexical categories [19, 23]. Categorisation is suggested to be critically enhanced when these phonological cues occur alongside other distributional information [2, 4, 55, 56]–with some studies showing that when categories are not marked by multiple corresponding cues, infants fail to learn them altogether [8, 19, 23] (see also [10, 97]).

Thus, we incorporated phonological cues to words (within-word harmony among consonants and vowels) and categories (within-category commonalities in phonological structure) into the artificial language, and tested learning using the same tasks as in Experiment 1. We expected to observe greater learning for both tasks than in Experiment 1, with participants drawing on phonological cues to word structure to help during segmentation, and phonological similarity between words in the same categories to help with categorisation. Further, we expected that learning would be best for the markers group, who received both types of cue. Because language proficiency, as measured by vocabulary size, was found to relate to patterns of looking in Experiment 1, we also included interactions between vocabulary size and the other conditions of the study in subsequent exploratory analyses.

### Method

#### Participants

Participants were 32 infants (10 boys, 20 girls), aged between 11.5 and 12.5 months (mean age = 368 days), recruited from Lancaster, Lancashire UK. All infants were monolingual native English learners, born at term, with normal vision and hearing, and were typically-developing at the time of testing. Infants were tested in the laboratory at Lancaster University. Two additional participants were tested but were excluded from analysis due to fussiness and experimenter error.

#### Design

The experimental design was identical to that used in Experiment 1 (*No Marker*: N = 16; boys = 7, girls = 9; *Markers*: N = 16; boys = 3, girls = 13).

#### Materials

The stimuli and materials were created in the same way as for Experiment 1, but with a critical distinction: the target words were created such that they contained phonological cues to word-hood and category membership (see Table 5). Specifically, *A words* were composed of front vowels and plosive consonants (i.e., *pebi*, *gide*, *kiti*), whereas *B words* contained back vowels and continuant consonants (i.e., *lumo*, *joru*, *wunu*). Thus, here, both the Markers and No Markers groups could conceivably distinguish the targets into their respective categories using this phonological information, but the Markers group will have an additional distributional cue to aid categorisation.

**Table 5:** Example speech streams for each condition in experiment 2 (with phonological cues).

	Target words	Marker words	Speech Stream Excerpt
No Markers	*pebe*, *gide*, *kiti*, *lumo*, *joru*, *wunu*		*…lumo-**pebe*-*gide**-joru-**kiti**-wunu-**gide**- lumo-**pebe**…*
Markers	*"*	***vi***, ***zhae***	*…****zhae****-lumo-****vi****-pebe-****vi****-gide-****zhae****- joru-****vi***-*kiti*-***zhae****-wunu-****vi****-gide-****zhae****- lumo-****vi***-*pebe**…*

*Note*. Items that are underlined belong to Category A, whereas items that are not underlined belong to Category B. Bold items with and without an underline indicate marker words for Category A and Category B words, respectively. Dashes indicate word boundaries, but these were not physically denoted in the continuous speech; items followed each other directly with no pauses between words.

#### Procedure

The procedure was identical to that used in Experiment 1.

## Results & discussion

### Data preparation

Filtering criteria were applied to the data in the same way as in Experiment 1.

### Segmentation

On average, infants looked to *word* trials for *M*_raw_ = 8596.588 ms (*SE*_raw_ = 589.586), and to *non-word* trials for *M*_raw_ = 8595.538 ms (*SE*_raw_ = 618.696).

As in Experiment 1, the looking times data were log transformed to rectify skewness (determined through visual inspection of histograms and QQ plots, and through the Shapiro-Wilk normality test; W = .798, *p* < .001), and Linear mixed effects analysis was performed on the data, modelling the probability of looking times considering variation across participants and materials, as well as across the two types of test items (words and non-words), to determine whether these differentially affected looking behaviour. The model was built following the same specifications as the analogous model in Experiment 1, with fixed effects and interactions for word type and markers condition, and an initial maximal random effects structure [74], with random intercepts of subject, trial (1–8), presentation version (A or B), stimuli location (left or right), and item, with a nested random slope of language version (1–4). If the model did not converge, then the random effects structure was simplified until convergence was no longer an issue. A summary of the final model is reported in Table 6.

**Table 6:** Summary of the final linear mixed-effects model of (log transformed) looking times on the segmentation trials for participants in experiment 2.

Fixed effects	Estimated coefficient	*SE*	Wald confidence intervals		*t* value	*p* value (*t*)
			2.50%	97.50%		
(Intercept)	8.808	.164	8.488	9.129	53.824	< .001
Word type	.002	.03	-.057	.061	.069	.945
Markers condition	-.005	.078	-.157	.147	-.065	.949
Word type * markers condition	.058	.03	-.001	.116	1.916	.057
Random effects	Variance	Std. Dev.				
Subject (Intercept)	.163	.404				
Subject: word_type (slope)	.0002	.013				
Trial (Intercept)	.166	.408				

236 observations, 32 participants, 8 trials. R syntax for the final model is: (logtotallooking ~ word_type*markers_condition + (1+word_type|subject) + (1|trial), data = seg_phono, REML = TRUE, control = lmerControl(optimizer = "bobyqa",optCtrl = list(maxfun = 1e+9))).

**Fig 4:**
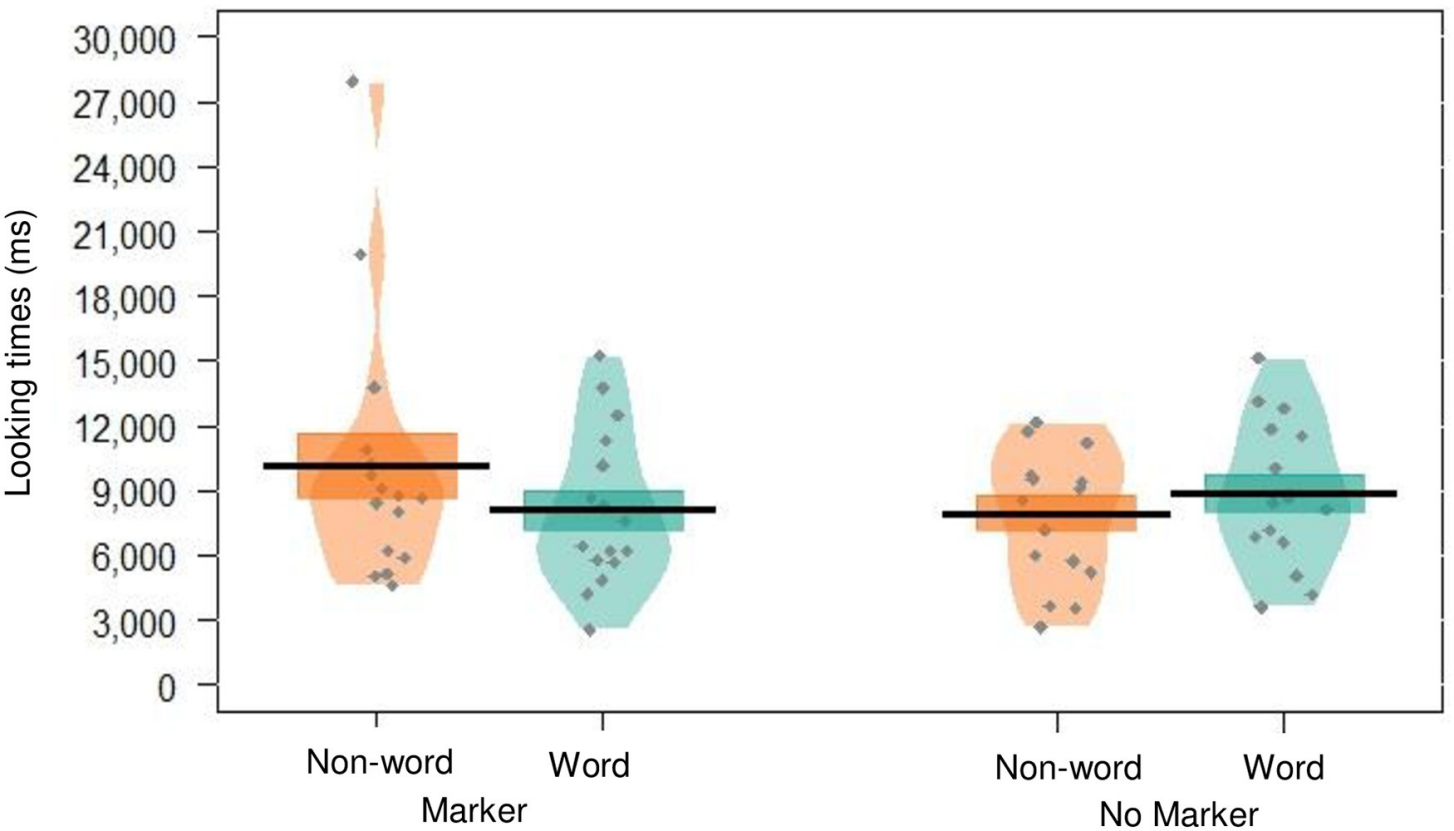
Pirate plot depicting the (raw) mean looking times to words and non-words, given for each condition. Black lines indicate the mean, and the coloured blocks indicate *SE*. The distribution of looking times is given for each group, with individual data points in grey. We note that subsequent supplementary exploratory analysis found that infants in the No Marker group were not homogeneous in their looking preferences–thus this visualisation combines data from two opposing preferences; a novelty preference (children with High CDI scores) and a familiarity preference (children with Low CDI scores); see Fig 5).

There was no significant effect of word type, with infants looking similarly to words and non-words overall (*p* = .945), and there was also no significant effect of markers condition (*p* = .949). However, the interaction between word type and markers condition was approaching significance (*p* = .057, *semi partial* $R_{m\;}^{2}=.114$), with trends in the means suggesting that infants attended differently to words versus non-words at test (indicating segmentation), with the direction of this difference being mediated by the presence/absence of markers in the training speech (see Fig 4).

The data suggest that infants may have been able to segment the speech into words, but were equally successful at doing so regardless of which training stream they received (as indexed by the similar differences in looking times to each type of trial). Thus, as in Experiment 1, the results indicate that high frequency words did not improve infants’ segmentation, though the presence of phonological cues perhaps did.

Interestingly though, the learning effects observed for the markers condition versus the control condition are distinct at the group level, with infants in the control condition displaying a familiarity preference (preferring words), whereas infants in the markers condition displayed a novelty preference (preferring non-words). This may be due to the different task demands that these conditions impose upon the learner; though non-words do not occur in either familiarisation stream, they may compete with words in slightly different ways for each group. For the control group, non-words are statistical competitors to words (since they could feasibly occur, but with lower transitional probabilities), whereas for the markers group non-words contain pairs of syllables that cannot conceivably appear together. Additionally, at test the markers group hear target words without their preceding markers for the first time, which may reduce the familiarity of these items. Together, these factors may mediate the complexity of the task for each group, and may have led to the different preferences that emerged at test. An alternative explanation is that this directional difference indicates that learning was indeed better for the markers condition–in line with the suggestion that a novelty preference reflects greater encoding, or a more mature response [60]. Higher powered replications are required to disentangle and confirm these possibilities.

#### Exploring looking preferences and vocabulary size: Segmentation

As in Experiment 1, we performed a subsequent exploratory analysis to examine learning as a function of language proficiency [57, 58, 60]. We performed a median split on the data according to infants’ receptive CDI scores, with infants scoring 63 or higher classified as *High-Vocabulary*, whereas those with a score of 62 or below were classified as *Low-Vocabulary*. We performed linear mixed-effects analysis on the data, with the critical tests being the interactions involving vocabulary size, word type, and markers condition. The models were built in the same way, and with the same random effects structure, as those described above. See Table 7 for a summary of the final model.

**Table 7:** Summary of the linear mixed-effects model of (log transformed) looking times on the segmentation trials for participants in experiment 2 (with median split for vocabulary size).

Fixed effects	Estimated coefficient	*SE*	Wald confidence intervals		*t* value	*p* value (*t*)
			2.50%	97.50%		
(Intercept)	8.808	.165	8.485	9.132	53.370	< .001
Word type	.1 x10^-3^	.03	-.06	.06	.004	.997
CDI score	-.077	.081	-.235	.081	-.96	.347
Markers condition	.008	.081	-.15	.166	.103	.919
CDI score*Word type	.056	.031	-.004	.116	1.830	.069
CDI score*Markers condition	-.021	.081	-.179	.137	-.265	.793
Word type*Markers condition	.052	.03	-.007	.112	1.718	.088
CDI score*word	.036	.031	-.025	.097	1.167	.245
type*Markers Condition						
Random effects	Variance	Std. Dev.				
Subject (Intercept)	.171	.414				
Subject: word type (slope)	.6 x10^-3^	.025				
Trial	.166	.408				

236 observations, 32 participants, 8 trials. R syntax for the final model is: (logtotallooking ~ cdi_score*word_type*markers_condition + (1+word_type|subject) + (1|trial), data = seg_phono, REML = TRUE, control = lmerControl(optimizer = "bobyqa",optCtrl = list(maxfun = 1e+9))).

**Fig 5:**
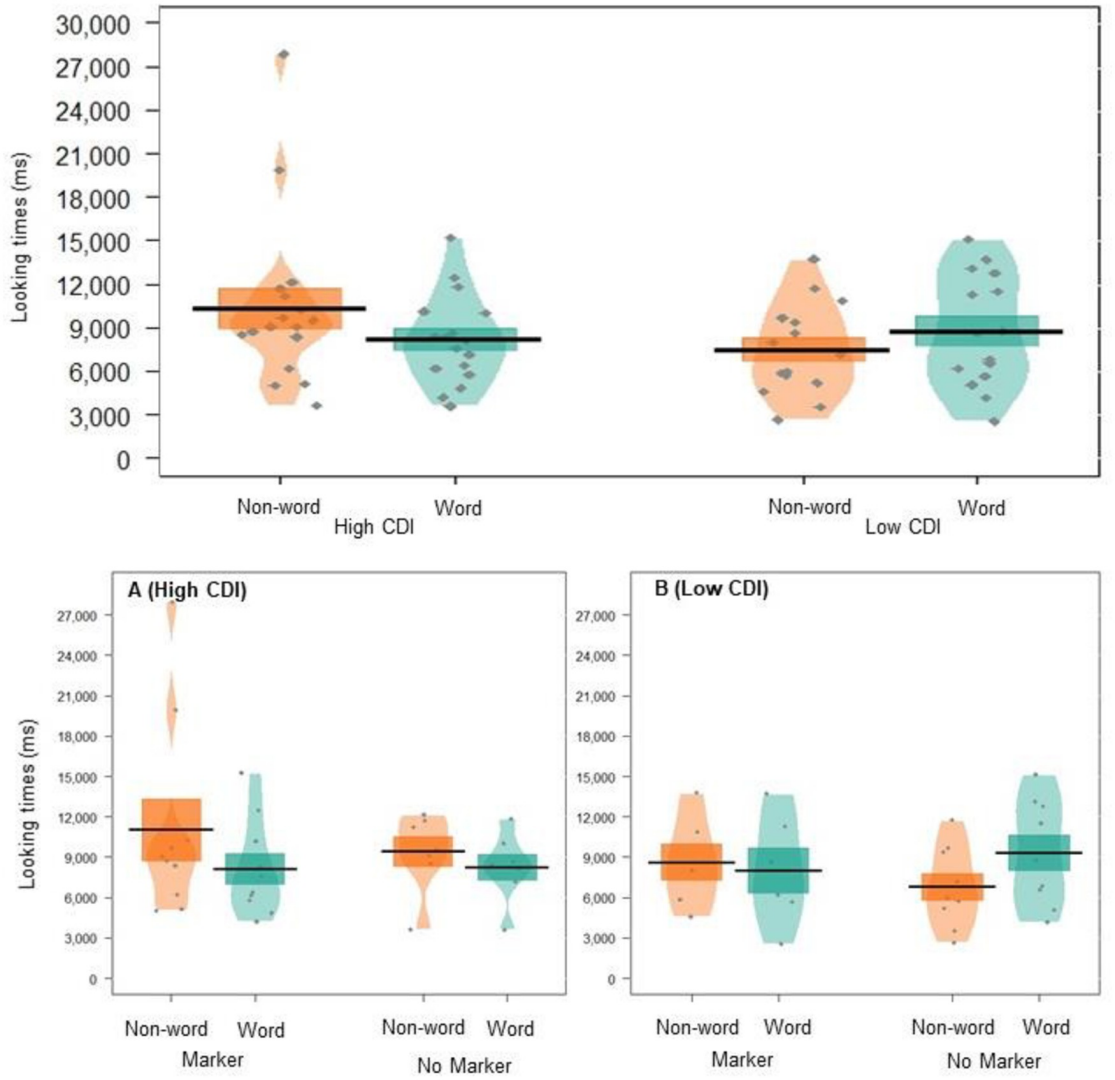
Pirate plot depicting the (raw) mean looking times to words and non-words for the participants in experiment 2. The top panel displays this data for High and Low CDI groups. The bottom panel breaks this down into each markers condition, with the High CDI group in panel A, and the Low CDI group in panel B. Black lines indicate the mean, and the coloured blocks indicate *SE*. Coloured shapes show the distribution of looking times for each group, with individual data points in grey.

There was no effect of vocabulary size on overall looking times (*p* = .347). However, as with Experiment 1, the interaction between vocabulary size and word type was approaching significance (*p* = .069, *semi partial* $R_{m\;}^{2}=.114$); high-vocabulary infants had a novelty preference, looking longer at non-words (*M*_raw_ = 9551.133, *SE*_raw_ = 911.181) than words (*M*_raw_ = 8238.647, *SE*_raw_ = 690.846), whereas low-vocabulary infants had a familiarity preference, looking longer at words (*M*_raw_ = 9027.444, *SE*_raw_ = 1002.683) than non-words (*M*_raw_ = 7589.649, *SE*_raw_ = 820.363, see Fig 5). This suggests that infants’ looking preferences were mediated by their language development in line with the results of Experiment 1, and provides further support for the notion that infants’ looking preferences are driven by their linguistic maturity [60]. The three-way interaction between CDI score, word-type, and markers group was not significant (*p* = .245).

### Categorisation

On average, infants looked to *consistent* trials for *M*_raw_ = 4586.94 ms (*SE*_raw_ = 314.29), and to *inconsistent* trials for *M*_raw_ = 4380.63 ms (*SE*_*r*aw_ = 254.83).

The data were log transformed in order to normalise the distribution (skewness was determined through visual inspection of histograms and QQ plots, and through the Shapiro-Wilk normality test; W = .844, *p* < .001). Linear mixed effects analysis was performed on the transformed data, and the model was fitted in the same way as the analogous model in Experiment 1; with fixed effects and interactions for trial type and markers condition, and a maximal random effects structure [74], with random intercepts of subject, trial (1–8), presentation version (A or B), stimuli location (left or right), and item, with a nested random effect of language version (1–4). The random effects structure was simplified until the model converged.

**Fig 6:**
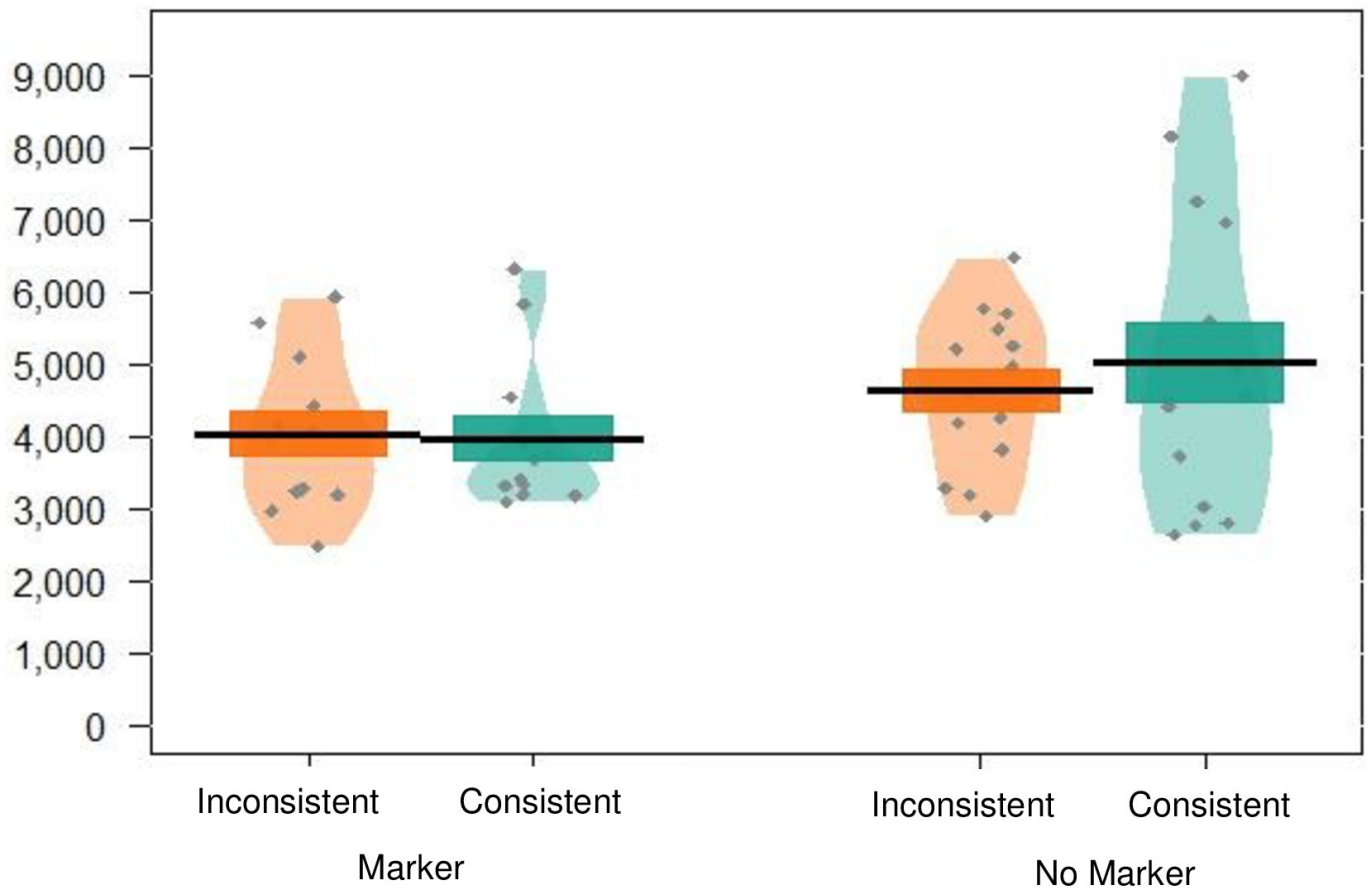
Pirate plot depicting the mean (raw) looking times to trials containing words from the same versus different categories for each condition. Black lines indicate the mean, and the coloured blocks indicate *SE*. The distribution of looking times is given for each group, with individual data points in grey.

There was no significant effect of trial type (*p* = .667), indicating that infants looked similarly to trials containing words from the same category and trials containing words from different categories. There was also no effect of markers condition (*p* = .149), and no significant interaction between trial type and markers condition (*p* = .97), suggesting that infants’ looking behaviour was not driven by their discrimination of similar versus different categorisation trials (see Fig 6, and see Table 8 for a summary of the final model).

**Table 8:** Summary of the linear mixed-effects model of (log transformed) looking times on the categorisation trials for participants in experiment 2.

Fixed effects	Estimated coefficient	*SE*	Wald confidence intervals		*t* value	*p* value (*t*)
			2.50%	97.50%		
(Intercept)	8.26	.0641	8.135	8.386	128.879	< .001
Trial type	.018	.042	-.064	.1	.432	.667
Markers condition	.069	.047	-.022	.161	1.492	.149
Trial type*Markers condition	.001	.04	-.076	.079	.037	.970
Random effects	Variance	Std. Dev.				
Subject (Intercept)	.018	.133				
Subect:word type (slope)	.002	.048				
Trial (Intercept)	.015	.124				

169 observations, 26 participants, 8 trials. R syntax for the final model is: logtotallooking ~ trial_type*markers_condition + (1+word_type|subject) + (1|trial), data = anchors12_cat_filt1500p, REML = TRUE, control = lmerControl (optimizer = "bobyqa", optCtrl = list (maxfun = 1e+9))).

#### Exploring looking preferences and vocabulary size: Categorisation

Supplementary exploratory analysis was performed on the categorisation data to examine whether infants’ looking behaviour was shaped by their linguistic maturity. Linear mixed effects analysis was performed on the data to test for differences in looking behaviour for high vs low vocabulary infants (using the same median split criteria, and the same random effects as structure described above). All effects and interactions involving CDI score were not significant (all *p* > .08); see the supplementary materials provided on OSF for an overview of the full model). There was thus no evidence that infants’ looking on the categorisation task was shaped by their linguistic maturity, or by the interaction between this and other variables.

## General discussion

High frequency words have been implicated as playing a key role in language acquisition, with prior research proving them to be advantageous for speech segmentation (e.g., [29, 31, 34, 42, 43]), and lexical categorisation [2, 9, 43, 45, 98]. We examined whether 12-month-old infants can draw on the effects of the same high frequency words to assist both of these tasks together during learning. In the studies presented here, there was no conclusive evidence to suggest that infants drew on the high frequency marker words during either segmentation or categorisation at the group level–contrary to our predictions.

Infants’ sensitivity to distributional information in speech is well established (e.g., [6]), as is their ability to use this information to help with the detection of word-boundaries (e.g., [7, 14, 29], see [99] for a meta-analytical review). However, in the present set of studies, infants’ capacity for statistical speech segmentation was lower than prior research might predict. In Experiment 1, at the group level, there was no evidence of speech segmentation, regardless of whether speech comprised target words only (mirroring classic studies of statistical segmentation), or target words plus high frequency markers–which we predicted would benefit learning. In Experiment 2, when the transitional probabilities were supplemented with phonological cues, infants’ segmentation was seen to improve, but high frequency words were not seen to help learning.

While speech segmentation effects at the group level were weak, in both Experiments 1 and 2 exploratory analyses revealed interesting effects at the individual level–providing some indication that participants distinguished between the different types of item at test, suggesting segmentation, and providing converging evidence to suggest that infants’ looking preferences were mediated by their linguistic maturity, with high- and low-vocabulary infants demonstrating novelty and familiarity preferences respectively. This is in line with the prior suggestion that infants’ looking preferences at test are dynamic [59], with infants switching from a familiarity to a novelty preference as a function of their linguistic maturity (e.g., [58, 60]). These data support the notion that infants’ looking behaviour can serve as a rich source of data, providing valuable insight into learning from an individual differences perspective, as well as at the group level. We note, though, that higher powered replications are necessary to confirm the nature of the marginal effects we report here.

The results suggesting that phonological cues promoted infants’ speech segmentation are intriguing, and are in keeping with Mintz et al.’s [35] demonstration that 7-month-old native English learning infants could use vowel harmony cues to segment new words from continuous speech. This is particularly noteworthy since infants in the studies at hand had no experience with vowel harmony in their native language–adding further support to Mintz et al.’s [35] suggestion that this cue may apply broadly during language learning in infancy (though see [100], for evidence suggesting sensitivity may be shaped by infants’ input statistics). These data can also be seen to provide support for the notion that phonological structure of words can be discerned from continuous speech (e.g., [73, 101–105])–indexing infants’ capacity to perform multiple tasks together during language acquisition (in this case, statistical speech segmentation, and phonological acquisition).

Although infants had an increased ability to identify target words when phonological cues supplemented transitional probabilities in speech, this ability was not significantly mediated by the presence of high frequency words–contrary to our expectation. This failure to replicate the *anchor effect* may in part be due to increased task complexity in the study at hand relative to previous research. Specially, the present studies used novel items as high-frequency words, which learners had to discover in speech along with targets, whereas most prior studies of the anchor word phenomenon used high frequency words that were already familiar to learners (e.g., [29]), or that became familiar to them in an initial training phase–before hearing the to-be-segmented speech (e.g., [34]). Our decision to refrain from including such a familiarisation phase was informed by the use of analogous words in natural language; determiners and pronouns seldom occur in isolation in single-word utterances (note that we focused on these words due to their potential joint impact on segmentation and categorisation; see e.g., [43], and see also [45]). However, this meant that infants were faced with the initial challenge of finding these items in the speech stream, which may have substantially increased the difficulty of the task [106, 107]. It is possible that greater benefits to segmentation would emerge once high frequency words have reached a certain threshold of familiarity (perhaps with prior exposure, or with a longer training stream), thereby facilitating the interplay between top-down and bottom-up processing suggested in prior research [33, 34] (see [45] for similar arguments). In future studies, measuring infants’ knowledge of the high frequency words separately (and perhaps relating this to their performance on the segmentation task) would give valuable insight into this possibility.

Relatedly, introducing unknown high-frequency marker words influenced the transitional probabilities between syllables in the speech stream. While young children are undoubtedly sensitive to variation in transitional probabilities [6], presenting high-frequency marker words along with bisyllabic targets reduced the difference among syllable-transition probabilities within versus between words. We did not distinguish between effects due to the presence of marker words and effects due to modulation of transitional probabilities precisely because these effects are related in natural language. However, in an artificial language learning paradigm such as this, where the high-frequency marker words are to be learned at the same time as the rest of the language, the reduction in transitional probability variation for the marker condition compared to the no-marker condition (marked by an increase in between-word TPs) may have reduced the potential for finding effects on segmentation.

Nevertheless, for the markers group, infants’ emerging ability to segment words from speech in Experiment 2 is noteworthy given the increased complexity of speech they heard (i.e., words of different lengths). Infants’ ability to recognise targets in the absence of the marker words at test is consistent with prior demonstrations that infants can segment around high-frequency marker words [28, 29, 31, 34, 42, 43]- though in this case they did not enhance learning.

There are several possible explanations as to why infants did not display stronger segmentation effects in line with those observed in prior research. First, in both Experiments, the speech stream comprised target words that were bisyllabic, yet many prior observations of statistical segmentation stem from studies using trisyllabic words (e.g., [6]) which may be easier to segment [50]. Second, for the markers condition, poor segmentation may have been due to the increased complexity of the speech; by design participants in the markers group received input containing two types of words—targets, which were bisyllabic to necessitate computation of within-word transitional probabilities, and markers, which were monosyllabic, to reflect the properties of high frequency function words in natural language [43]. Such variation permits crucial examination of how processing of high frequency function words interacts with the computation of word-internal transitional statistics during learning. However, the difference in word length may have made it difficult for infants to break into the speech stream, particularly in Experiment 1: Prior research has demonstrated that segmenting speech under such conditions is challenging (e.g., [30]), and perhaps impossible without additional scaffolding (e.g., [51, 108]), attested to here by the improvements seen in Experiment 2, when the distributional cues are supplemented with additional phonological regularities. Indeed, these data speak to the key possibility that, when faced with such complexity, very brief exposure to transitional information alone may be insufficient for segmentation–with learners drawing on the many additional cues in language to overcome these difficulties ‘in the wild’ (see e.g., Frost and Monaghan [109] for discussion on the way in which statistical regularities work in tandem with other cues during language acquisition).

Another possibility is that the studies at hand did not have sufficient experimental power to observe an effect (see Black and Bergmann [99], for recommended sample sizes for replicating segmentation effects relating to those found by Saffran et al., [6]). Of note is that the differences observed on the segmentation task in Experiment 2 are of a similar magnitude to those seen in comparable studies, but with a greater degree of variation around the means (e.g., [6]). Thus, it is possible that these data captured an emerging effect, but that greater power would be necessary to see robust results. Implementing this in subsequent research will help confirm the nature of the effects seen here.

We have described the measure of infants’ preference for words versus non-words as a segmentation test, however it remains a possibility that performance could be driven by sequence familiarity. Equally, learning from the languages with and without marker words could have proceeded rather differently, with marker words being considered as either part of the target word (e.g. an affix; c.f., PARSER [104]), or as function words that mark the word’s role (c.f., the PUDDLE model [43], see Frost et al., 2019 for similar arguments). Future research examining infants’ preference for sequences that include and omit marker words alongside targets would enable us to unpack these alternatives (for similar suggestions, see Frost et al., [45]). While these are important considerations, the results seem to indicate that 12-month-olds could discriminate between target words (either as an isolated word, or as the root of a word that appears at test without its prefix) and sequences that comprise two portions of different words–but only when the training language comprised phonological cues in addition to transitional probability statistics.

Although there was some evidence to suggest that infants could segment the speech into words under certain conditions, there was no evidence that infants could use the marker words to inform categorisation. In a related paradigm, Frost et al. [45] found that adults could discern targets into distributional categories when they occurred alongside category-denoting marker-words in speech. Relatedly, Lany [9] found that much older infants could form lexical categories based on novel determiner-noun co-occurrence, with these categories influencing their subsequent labelling of items in different semantic categories (animals/vehicles). However, these findings were not conceptually replicated in the study at hand–even when co-occurrence statistics were supplemented with additional phonological cues. There are a number of possible explanations as to why this may have been the case.

One possibility is that infants are unable to make use of distributional cues to inform categorisation (in this case, high frequency function words, and the correlation between these and phonological cues)–however this seems unlikely given the wealth of prior evidence to the contrary (e.g., [8, 9, 23, 110], see also Monaghan et al., [4]). A more plausible explanation is that infants’ poor segmentation performance impacted their ability to discover the categories that were contained within the speech; in prior studies of distributional categorisation, learners received segmented words in short utterances, meaning task complexity was substantially reduced relative to the study at hand. Here, infants had to first segment the items, then compute over their regularities to form categories. Thus, we expect that should segmentation be seen to improve, so too would categorisation.

Another possibility is that infants in our study had not yet developed the requisite skills to discern distributional categories from speech. Although there are demonstrations of this ability for 12-month-old infants (e.g., [19, 23, 111, 112]) many of the observations of this effect are for older infants, with the majority of studies reporting effects for children in their second year of life–typically between 17 months [8] and 22 months old [9, 10, 97]. Lany and Saffran [97] noted that while 22-month-olds could use distributional cues to inform semantic categorisation, infants used different learning strategies depending on their linguistic proficiency–with only more advanced infants (indexed by high MCDI scores) drawing on distributional cues relating to co-occurrence, while infants with smaller vocabularies relied more on phonology. Similarly, Lany [9] found evidence for distributional categorisation for only 22-month old infants who scored highly on the grammar index of the MCDI. Taken together, these results suggest this ability may build over development, and may not yet have emerged in our sample of 12-month-olds. Replications with older infants will shed light on this possibility.

We also note that prior demonstrations of related effects with infants used languages which marked category membership with phonological cues concerning word-length (i.e., with words in each category having a different number of syllables), rather than phonotactics. Though infants did show sensitivity to these phonotactic cues in the study at hand (indicated by the boost to segmentation in Experiment 2), it is possible that they were more difficult to draw upon for categorisation than the word-length cues used in prior research.

In sum, prior studies have documented infants’ remarkable aptitude for computing over the distributional properties of linguistic input for speech segmentation and lexical categorisation, and recent research has suggested that the same high-frequency words may prove useful to both of these tasks [43]. However, there was no evidence to suggest that this was the case for 12-month-old infants in the study at hand–with no significant benefit observed for either segmentation or categorisation. That is not to say that high frequency words do not assist early language acquisition altogether: We suggest that for this benefit to emerge, high frequency words must attain a critical threshold of familiarity–possibly through a combination of highly frequent exposure, and appearing in isolation or at utterance/phrasal boundaries. Importantly, our results do indicate that phonological cues may provide a useful scaffolding for statistical speech segmentation, offering key support for the role of these cues in language acquisition. These data can also be seen to provide converging evidence that infants’ looking preferences at test are meaningful, and may serve as a rich source of individual differences data–with the direction of infants’ looking preferences here relating to the linguistic maturity of the learner.
